# Variations in SARS-CoV-2 Spike Protein Cell Epitopes and Glycosylation Profiles During Global Transmission Course of COVID-19

**DOI:** 10.3389/fimmu.2020.565278

**Published:** 2020-09-04

**Authors:** Wenxin Xu, Mingjie Wang, Demin Yu, Xinxin Zhang

**Affiliations:** ^1^Department of Infectious Diseases, Research Laboratory of Clinical Virology, National Research Center for Translational Medicine, Ruijin Hospital, Shanghai Jiao Tong University School of Medicine, Shanghai, China; ^2^Department of Digestive Diseases, Ruijin Hospital North, Shanghai Jiao Tong University School of Medicine, Shanghai, China; ^3^Clinical Research Center, Ruijin Hospital North, Shanghai Jiao Tong University School of Medicine, Shanghai, China

**Keywords:** SARS-CoV-2, spike protein, cell epitope, glycosylation, variation

## Abstract

Coronavirus disease 2019 (COVID-19), caused by severe acute respiratory syndrome coronavirus 2 (SARS-CoV-2), has led to an outbreak of a pandemic worldwide. For better understanding the viral spike (S) protein variations and its potential effects on the interaction with the host immune system and also in vaccine development, the cell epitopes, glycosylation profile and their changes during the global transmission course were characterized and compared with SARS-CoV for their glycosylation profile. We analyzed totally 7,813 sequences screened from 8,897 whole genome sequences on GISAID database up to April 26, and 18 S protein amino acid variations with relatively high frequency (≥10^−3^) were identified. A total of 228 sequences of variants had multiple variations, of note, most of them harboring the D614G mutation. Among the predicted 69 linear B cell epitopes, 175 discontinuous B cell epitopes and 41 cytotoxic T lymphocyte epitopes in the viral S protein, we found that the protein structure and its potential function of some sites changed, such as the linear epitope length shortened and discontinuous epitope disappeared of G476S. In addition, we detected 9 predicted N-glycosylation sites and 3 O-glycosylation sites unique to SARS-CoV-2, but no evidently observed variation of the glycan sites so far. Our findings provided an important snapshot of temporal and geographical distributions on SARS-CoV-2 S protein cell epitopes and glycosylation sites, which would be an essential basis for the selection of vaccine candidates.

## Introduction

The emergence of coronavirus disease 2019 (COVID-19), caused by severe acute respiratory syndrome coronavirus 2 (SARS-CoV-2), has led to an outbreak of a global pandemic (1). As no specific anti-viral therapies or vaccines have been developed yet, there is an urgent need for research regarding the structure and function of proteins of the virus.

SARS-CoV-2 is a single, positive stranded RNA virus, which codes for ORF1a, ORF1b, Spike (S), ORF3a, ORF3b, Envelope, Membrane, ORF6, ORF7a, ORF7b, ORF8, ORF9b, ORF14, Nucleocapsid, and ORF10 proteins. The S protein is a class I viral fusion protein. It is composed of an S1 subunit that binds to the host cell receptor, and an S2 subunit that mediates the fusion of the viral cellular membrane and is important for cell adhesion and the induction of protective immunity (2). Thus, the efficacy of neutralizing antibodies targeting the S protein may be affected by variations in its sequence (3). So far, the evolutionary pattern of S protein during the epidemic course remains unclear.

Several clinical studies have confirmed that host immune response was involved COVID-19 pathogenesis. Researchers detected IgM antibodies in 73% of patients and IgG in 54% of patients at day 14 after disease onset in COVID-positive patients (4). While circulating SARS-CoV-2-specific CD8^+^ and CD4^+^ T cells were identified in 70 and 100% of convalescent patients with COVID-19, respectively (5). The prediction of T and B cell epitopes in the S protein would provide valuable information for the development of vaccines and antibody-based therapeutics due to their dominant and long-lasting immune response. Viral S protein is modified by glycosylation, which may be implicated in immune evasion from the host immune system, shielding the protein surface from detection by antibodies, and affects the ability of the host to mount an effective adaptive immune response (6, 7). Indeed, the recently reported cryo-EM structure of SARS-CoV-2 suggested that, like SARS-CoV, the S protein of SARS-CoV-2 is also extensively glycosylated (8).

Therefore, investigating the glycosylation pattern and cell epitopes of the viral S protein may help understand its interaction with the host immune system and accelerate vaccine development. However, immunological information available on SARS-CoV-2 is lacking. In the present study, we compared the glycosylation of the S proteins of SARS-CoV-2 and SARS-CoV. Furthermore, we identified the variations in glycosylation and cell epitopes of SARS-CoV-2 during the global transmission course, and further explored the significance of these changes.

## Materials and Methods

### Sequence Analysis

SARS-CoV-2 genomic sequences submitted to Global Initiative of Sharing All Influenza Data (GISAID) database (https://www.gisaid.org/) till April 26, 2020, that met the following criteria, were retrieved: (1) whole genome sequence; (2) sequence length >29,000 bp; and (3) high coverage. Totally, 8,897 SARS-CoV-2 whole genome sequences were collected.

To identify the S gene of each viral strain, the viral genomes were aligned to the S gene of reference sequence (SARS-CoV-2 virus isolate Wuhan-Hu-1, MN908947) using BLAST (9), and sequences with unspecified bases (N) in the S gene were filtered out. Sequence manipulations were carried out using in-house Perl scripts. Further screening of SARS-CoV-2 nucleotide sequences was conducted according to the transmission course (December 2019–April 2020) and regions (Asia, Europe, North America, South America and Oceania) the samples were collected in.

The filtered SARS-CoV-2 S gene sequences were translated to amino acid sequences and aligned by using MEGA X software (10). Amino acid variations with frequency higher than 10^−3^ were detected by using the QAP software and defined as relatively high frequency variations (11). All sequences selected were classified by transmission course and region. In addition, the strains with multiple variations were also analyzed.

### Cell Epitope Prediction

Linear B cell epitopes for SARS-CoV-2 S protein (wild type and variant) were determined in BepiPred (http://www.cbs.dtu.dk/services/BepiPred), which combines a hidden Markov model and a propensity scale method. Briefly, protein sequences were uploaded in FASTA form, and score threshold for the epitope was set to 0.35. So those residues with scores above 0.35 were predicted to be part of an epitope. Discontinuous epitopes were predicted by the DiscoTope method (http://tools.immuneepitope.org/discotope/), we used 6VSB PDB as a 3D structure model of the wild-type SARS-CoV-2 S protein (). For variant S protein, we modeled a 3D structure of S protein harboring each amino acid variation.

Cytotoxic T lymphocyte (CTL) epitopes for the S protein of the SARS-CoV-2 reference sequence were identified with TepiTool from the Immune Epitope Data-base and Analysis Resource (IEDB) analysis resource (http://tools.immuneepitope.org/tepitool/) using the recommended method (12). After submitting protein sequences, we selected a panel of the 27 most frequent alleles in the global population and “moderate number of peptides” (8, 9, 10, and 11 mers) for prediction. IEDB recommended was chosen as the prediction method. CTL epitopes obtained were further confirmed using the NetMHCpan-4.0 server (http://www.cbs.dtu.dk/services/NetMHCpan-4.0/), which utilizes ANN trained on quantitative binding data and mass spectroscopy-derived major histocompatibility complex (MHC) eluted ligands to predict epitope-MHC-I binding (13). We selected the 12 most frequent human leukocyte antigen (HLA) class I alleles in the worldwide population (14), including HLA-A^*^01:01, HLA-A^*^02:01, HLA-A^*^03:01, HLA-A^*^11:01, HLA-A^*^23:01, HLA-A^*^24:02, HLA-B^*^07:02, HLA-B^*^08:01, HLA-B^*^35:01, HLA-B^*^40:01, HLA-B^*^44:02, and HLA-B^*^44:03. The rank thresholds for strong and weak binding peptides were 0.5 and 2%, respectively. The effects of corresponding amino acid variations on MHC class I binding levels were compared.

### Glycosylation Site Analysis

To identify significant differences in glycosylation patterns, N-linked glycosylation sites of SARS-CoV-2 and SARS-CoV were analyzed using the relevant web server (http://www.cbs.dtu.dk/services/NetNGlyc), we chose 0.5 as the predication threshold. The results were shown as a graphic illustrating the potential glycosylation sites. O-linked glycosylation sites were also predicted using NetOGlyc (version 1.0) to examine sequence context. The glycosylation sites of the two viruses were compared based on the transmission course and region, and the characteristics of variations were analyzed.

A 3D protein structure of SARS-CoV-2 S protein was obtained from the SWISS-MODEL server (swissmodel.expasy.org) using protein homology modeling, based on a cryo-EM structure (PDB ID: 6VSB) (15). To structurally model SARS-CoV-2 S protein glycosylation, all predicted O- and N-linked glycosylation sites were introduced into the crystal structure using the PyMol program (version 2.4.3). Different colors were used to mark several essential parts to help understand this more intuitively.

## Results

### Basic Information on All Sequences

#### Temporal and Regional Distributions of Sequences

**Figure 1:**
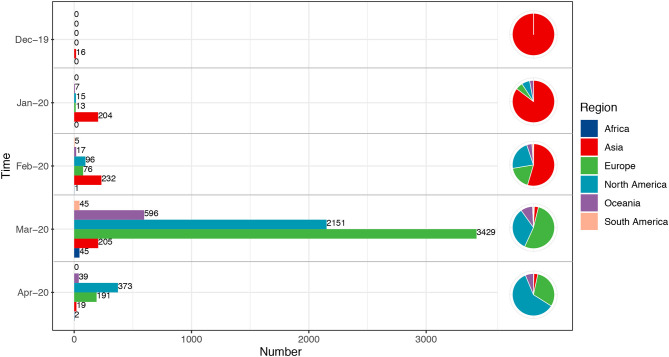
Temporal and geographical distributions of all sequences. All the sequences selected in this study were classified according to the transmission course and region. Time referred to the collection date of the samples, which were divided into groups by month from December 2019 to April 2020. Each group included the total number of all sequences in related month. Regions were classified by continent, including Africa, Asia, Europe, North America, South America, and Oceania. Number referred to the number of sequences. The sequences from December 2019 to February 2020 were concentrated in Asia, and the subsequent cases in March and April were mainly in Europe and North America.

According to the filtering criteria, finally 7,813 full-length S gene sequences of SARS-CoV-2 and 96 S gene sequences of SARS-CoV were retrieved. The glycosylation analysis of these two viruses was conducted using these sequences, while cell epitope prediction was based on the reference sequence. All sequences were classified to explore whether SARS-CoV-2 had a special evolutionary pattern or variation mode during the global transmission course (Figure 1). Consistent with previous results, the sequences from cases from December 2019 to February 2020 were concentrated in Asia, and the subsequent sequences in March and April were spread in Europe and North America. The total number of sequences detected in March was much higher than that in January and February.

#### Amino Acid Variations of SARS-CoV-2 Spike Protein

**Figure 2:**
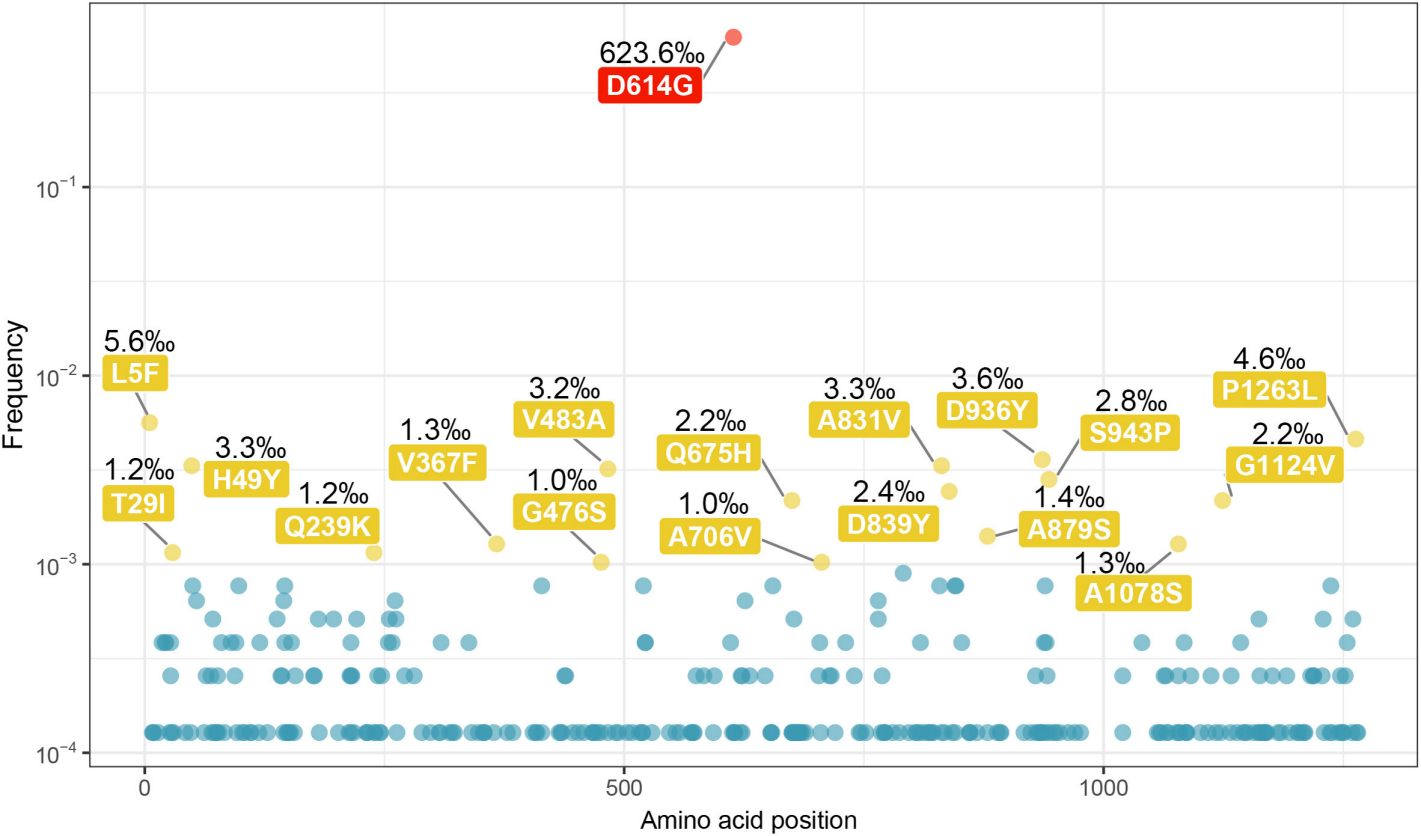
Amino acid variations on the sequences of SARS-CoV-2 spike protein. All the amino acid variations on the sequences of SARS-CoV-2 S protein were selected out, and the scatter plot was made according to the frequency of variation and the position on sequence. A total of 18 relatively high frequency sites were screened out (frequency > 10^−3^), including a highest frequency substitution site D614G. The sites with the highest frequency are indicated in red, and the rest of 17 were indicated in yellow.

**Figure 3:**
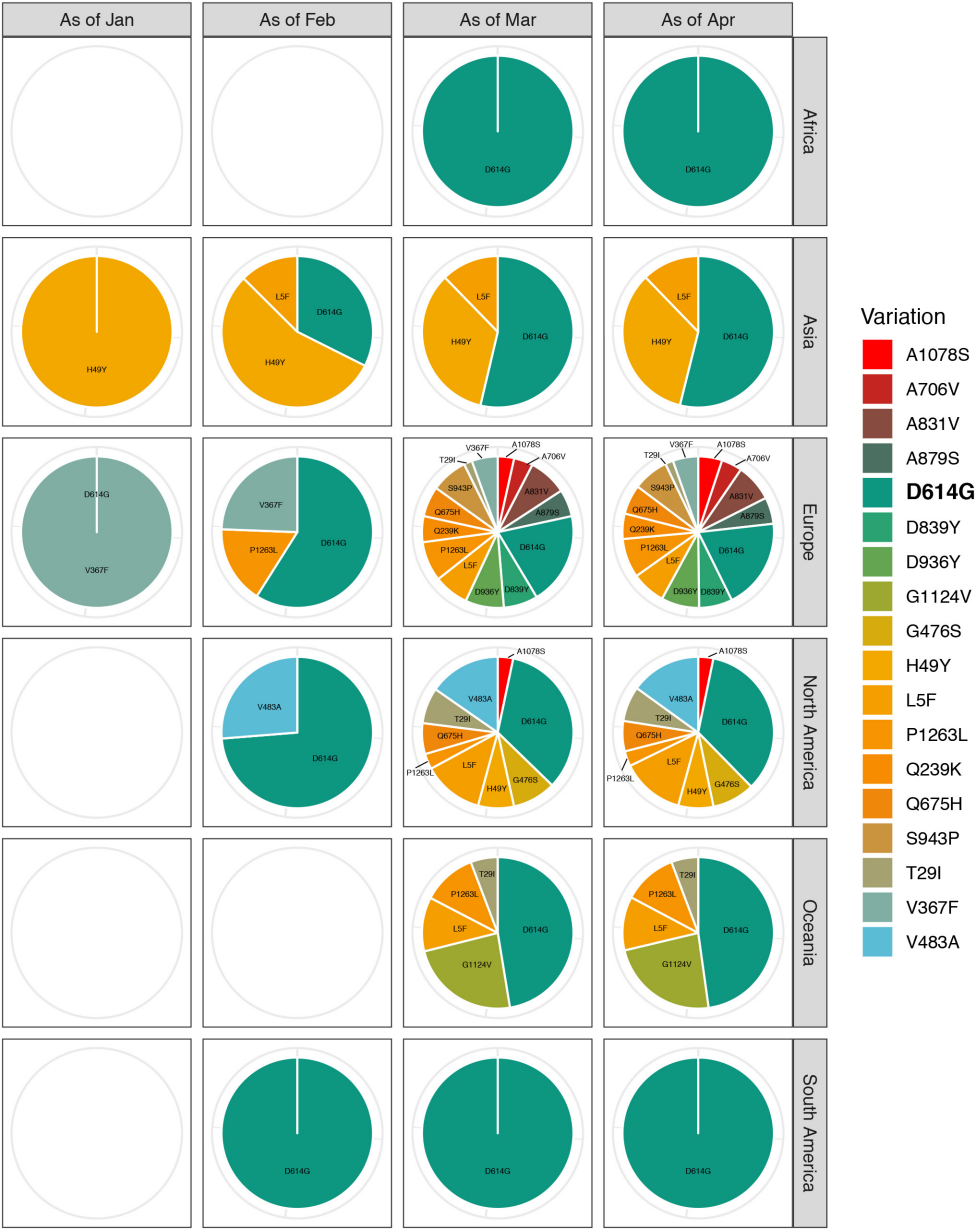
Temporal and geographical distributions of sequences with relatively high-frequency amino acid variations on SARS-CoV-2 spike protein. All the relatively high-frequency sequences selected, containing amino acid variations, were classified according to the transmission course and region. Time refers to the collection date of the samples containing amino acid variations, which is divided into groups by month from December 2019 to April 2020. Each group is the total number of sequences up to the end of this month. Regions are classified by continent, including Africa, Asia, Europe, North America, South America, and Oceania. The highest frequency substitution site D614G widely distributes around the world and spreads through the whole transmission process. With the proceeding of transmission course, the variety of amino acid variations in each continent is increasing. Especially, Europe has the most kinds of amino acid variants, some of which are unique to the region.

We generated a scatter diagram to analyze amino acid variations in S protein for the selected sequences (Figure 2). A total of 18 relatively high frequency amino acid variation sites were detected. The temporal and geographical distributions of the sequences containing amino acid variations above were comprehensively analyzed (Figure 3) and a detailed report regarding these distributions was summarized (Table S1).

Among the 18 amino acid variation sites, the highest frequency substitution was D614G. This variant was globally distributed and propagated throughout the timeline of viral transmission (first appeared in Zhejiang on January 24, 2020). This indicated a more aggressive feature that made it spreading more quickly (16). We observed that most remaining sites had their special temporal and regional propensities, such as Q239K, A831V, D839Y, A879S, D936Y, S943P, and P1263L, which were mainly concentrated in Europe in March, 2020. There was an increasing tendency in the number of the variety of variants in the sequence as the spread of the virus continued; Europe had the most kinds of amino acid variants, some of which were unique to the region.

**Figure 4:**
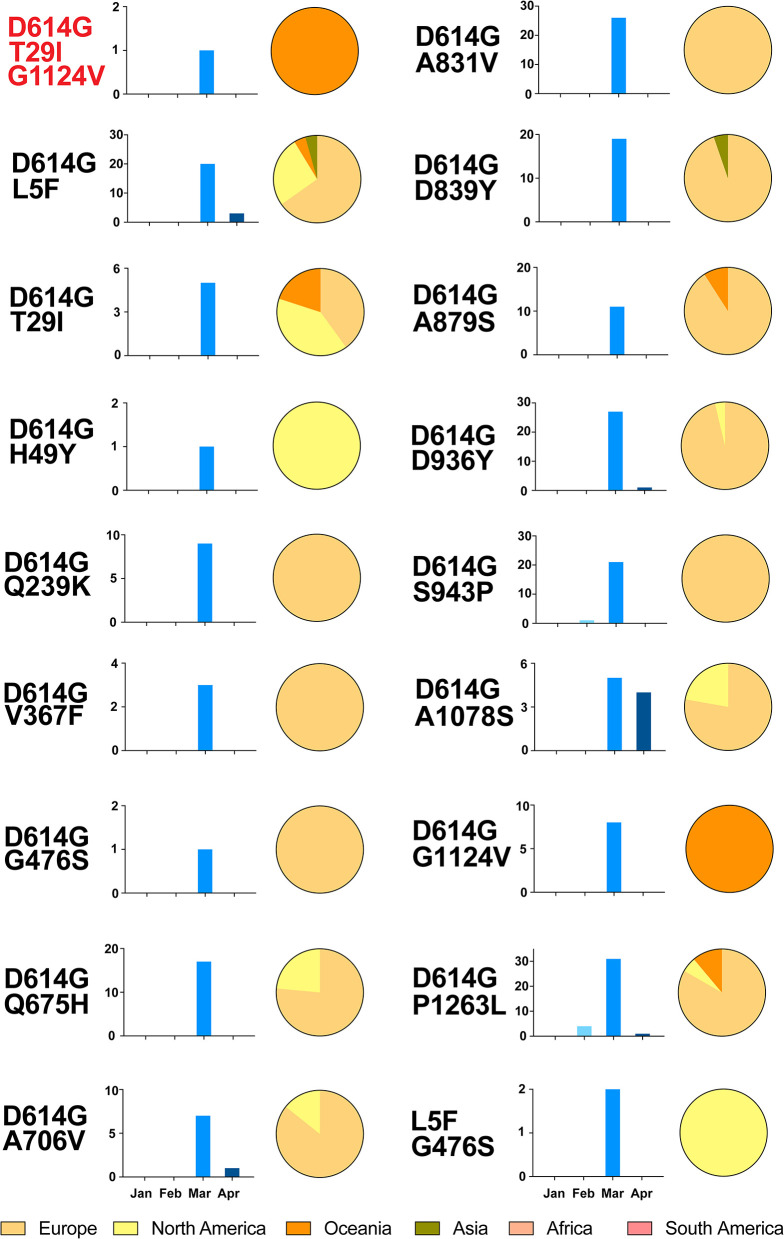
Temporal and geographical distributions of the sequences of variants with multiple relatively high-frequency amino acid variations on SARS-CoV-2 spike protein. A total of 18 kinds and 228 sequences of variants had multiple relatively high frequency variations. All of them had two variations, except one with T29I, D614G, and G1124V. These sequences were classified by the temporal and geographical distributions. Time refers to the collection date of the sequences from samples, using histogram (blue), which is divided into groups by month from January to April 2020. Regions are classified by continent, using pie graph (yellow), including Africa, Asia, Europe, North America, South America, and Oceania. It is obvious that most sequences with multi-variations were from Europe, and no one in South America and Africa. As for the temporal distribution, the majority of these were concentrated in March 2020, but none was detected in January.

Unexpectedly, variants with multiple relatively high frequency variations were found in 228 sequences (Figure 4 and Table S3). All contained two the more frequency variations, a total of 18 types, except for one triple variations (T29I, D614G, and G1124V). Remarkably, most of them harbored with D614G mutation (226 of 228). It is obvious that most sequences with multi-variations were from Europe, and no one was in South America and Africa. Regarding the temporal distribution, the majority of these were concentrated in March 2020, but none was detected in January.

### Cell Epitope Predictions of SARS-CoV-2 Spike Protein and Its Variations

#### B Cell Epitopes

Altogether, 69 peptides of S protein were predicted as linear B cell epitopes (Figure S1 and Table S2). Among them, amino acid substitutions T29I, G476S, D936Y, S943P, and P1263L changed the length of the linear B cell epitopes, whereas V483A, Q675H, and A706V had no influence on the length of linear epitopes, and the others had no effect on linear B cell epitopes (Table 1). Notably, two amino acid substitutions within receptor-binding domain (RBD) had different impacts. G476S shortened epitope length from 13 to 11 amino acids (YQAGSTPCNGAEG to YQASSTPCNGV), whereas V483A remained the same. D936Y nearly abolished the original epitope (DSLSST, 936–941), whereas S943P induced the formation of a novel 9 amino acid epitope (DSLSSTAPAL, 936–945).

**Table 1:** Variations in linear B cell epitope prediction of SARS-CoV-2 spike protein and its impacts.

**No**.	**Variation**	**Wide type epitopes**		**Variant epitopes**	
		**Length (start-end)**	**Peptides**	**Length (start-end)**	**Peptides**
1	T29I	11 (21–31)	RTQLPPAYTNS	7 (21–27)	RTQLPPA
2	G476S	13 (473–485)	YQAGSTPCNGAEG	11 (473–483)	YQASSTPCNGV
3	V483A	13 (473–485)	YQAGSTPCNGAEG	13 (473–485)	YQAGSTPCNGAEG
4	Q675H	13 (675–687)	QTQTNSPRRARSV	13 (675–687)	HTQTNSPRRARSV
5	A706V	4 (704–707)	SVAY	4 (704–707)	SVVY
6	D936Y	6 (936–941)	DSLSST	1 (939–939)	/
7	S943P	1 (943–943)	/	9 (936–945)	DSLSSTAPAL
8	P1263L	10 (1,256–1,265)	FDEDDSEPVL	6 (1,256–1,261)	FDEDDS

Using 3D structures of SARS-CoV-2 S protein, 175 discontinuous B cell epitopes were predicted (Table S2). Among the 18 substitution sites, only sites 476 and 706 were in discontinuous epitopes (Table 2). By construction of the 3D structures of S protein with the amino acid substitutions, we found that G476S substitution rendered the peptide as a non-epitope and A706V had no effect on epitope prediction.

**Table 2:** Variations in discontinuous B cell epitope prediction of SARS-CoV-2 spike protein and its impacts.

**No**.	**Position**	**Wide type**	**Variant type**	**Variation impact**
1	476	Gly	Ser	Epitope disappear
2	706	Ala	Val	Epitope unchanged

#### T Cell Epitopes

To assess the effects of amino acid substitution on CTL epitopes of SARS-CoV-2 S protein, we first predicted epitopes of “wild type” S protein based on the 27 most frequent alleles in the global population because there is significant overlapping among different HLA class I alleles. We further confirmed CTL epitopes with the 12 most frequent HLA class I alleles in the population according to a previous report (17). Here, we identified 41 peptides as strong binders, indicated by a % rank below the specified threshold of 0.5% (Table S2). For variant S proteins, T29I, H49Y, Q239K, V367F, V483A, A706V, A831V, D839Y, S943P, A1078S, and P1263L were located in the predicted CTL epitopes according to the HLA class I allele (Table 3). In contrast, G476S, D614G, Q675H, A879S, D936Y, and G1124V were outside of the CTL epitopes. Binding level results of T29I, V367F, A706V, and A831V demonstrated that these substitutions had low binding affinity in HLA-A01:01, HLA-B07:02, and HLA-B35:01 compared to the wild type, while H49Y, Q239K, V483A, D839Y, S943P, A1078S, and P1263L substitutions still had strong binding affinity with HLA molecules.

**Table 3:** Variations in cytotoxic T lymphocyte cell epitope prediction of SARS-CoV-2 spike protein and its impacts.

**No**.	**Variation**	**Bind level**											
		**HLA-A01:01**	**HLA-A02:01**	**HLA-A03:01**	**HLA-A11:01**	**HLA-A23:01**	**HLA-A24:02**	**HLA-B07:02**	**HLA-B08:01**	**HLA-B40:01**	**HLA-B35:01**	**HLA-B44:02**	**HLA-B44:03**
1	T29I	S→ W	/	/	/	S→ S	S→ S	S→ W	/	/	S→ W	/	/
2	H49Y	/	/	S→ S	/	/	/	/	/	/	/	/	/
3	Q239K	/	/	/	/	/	/	/	S→ S	/	/	/	/
4	V367F	/	/	/	/	/	/	/	/	/	S→ W	/	/
5	V483A	/	/	/	/	/	/	/	/	/	S→ S	/	/
6	A706V	S→ S	/	/	/	/	/	/	/	/	S→ W	/	/
7	A831V	S→ W	/	S→ S	S→ S	/	/	/	/	/	/	S→ S	S→ S
8	D839Y	/	/	/	/	/	/	/	S→ S	/	/	/	/
9	S943P	/	/	S→ S	S→ S	/	/	/	/	/	/	/	/
10	A1078S	/	/	/	S→ S	/	/	/	/	/	/	/	/
11	P1263L	/	/	/	/	/	S→ S	S→ S	S→ S	/	/	/	/

*HLA, human leukocyte antigen; S, strong binder; W, weak binder; /, no predicted T cell epitope; S→ W represents decreased binding affinity after variation; S→ S represents no change in binding affinity*.

### Glycosylation Site Analysis of SARS-CoV-2 Spike Protein and Comparison With SARS-CoV

**Figure 5:**
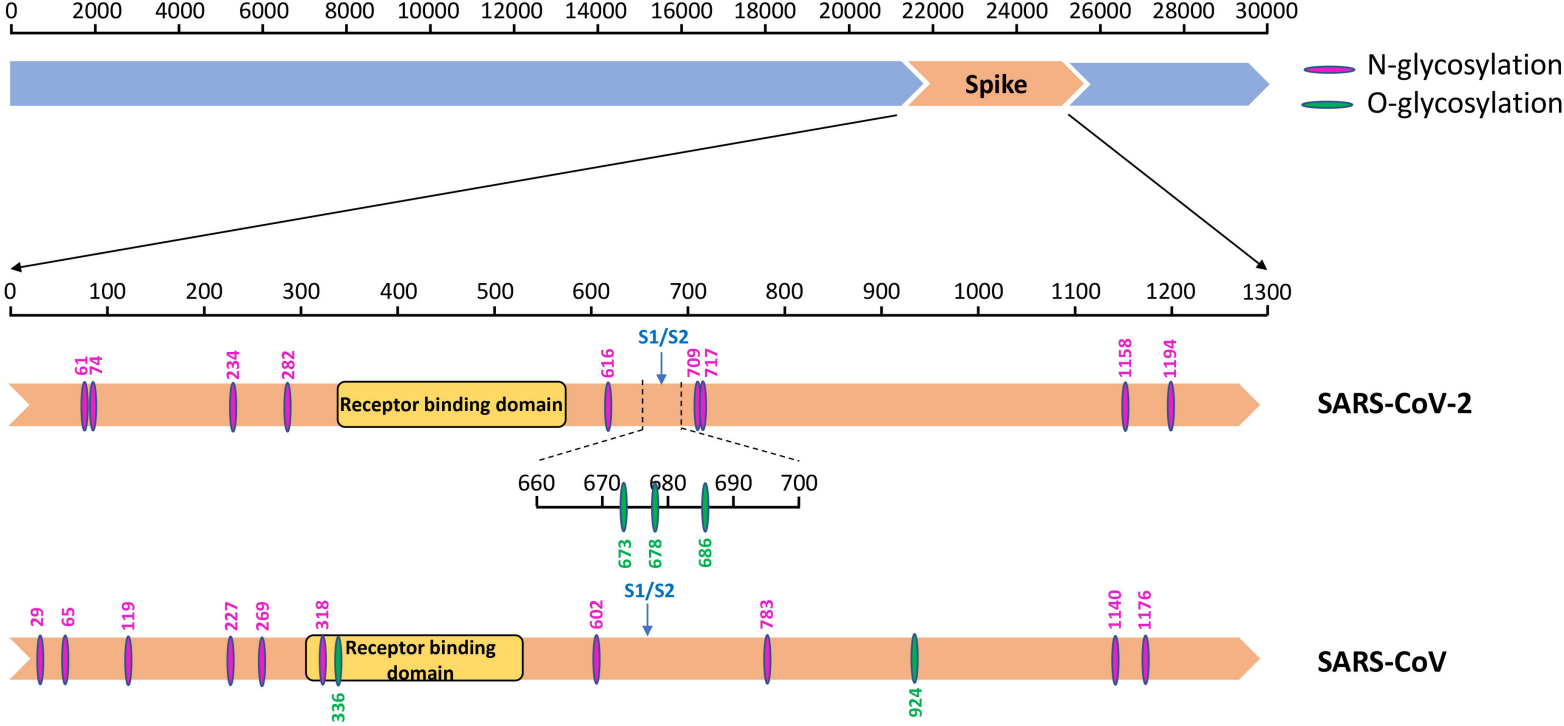
The glycosylation sites on the spike protein of SARS-CoV-2 and SARS-CoV. Diagram showing the S protein (orange) of SARS-CoV-2 and SARS-CoV with different functional domains indicated, and N- (amaranth) and O- (green) glycosylation sites.

To explore the characteristic glycosylation pattern of the viral S protein in both viruses, we first detected all N- and O-glycosylation sites on the SARS-CoV-2 and SARS-CoV. This data was used for comparative analysis (Table 4 and Figure 5).

**Table 4:** Comparison of N- and O-glycosylation sites on spike proteins between SARS-CoV-2 and SARS-CoV.

**SARS-CoV-2**					**SARS-CoV**				
**N-linked**			**O-linked**		**N-linked**			**O-linked**	
**Position**	**Sites**	**Subunit**	**Position**	**Subunit**	**Position**	**Sites**	**Subunit**	**Position**	**Subunit**
61	NVTW	S1	673	S1	29	NYTQ	S1	336	S1
74	NGTK	S1	678	S1	65	NVTG	S1	924	S2
234	NITR	S1	686	S2	119	NSTN	S1		
282	NGTI	S1			227	NITN	S1		
616	NCTE	S1			269	NGTI	S1		
709	NNSI	S2			318	NITN	S1		
717	NFTI	S2			602	NCTD	S1		
1158	NHTS	S2			783	NFSQ	S2		
1194	NESL	S2			1140	NHTS	S2		
					1176	NESL	S2		

#### SARS-CoV-2 vs. SARS-CoV

**Figure 6:**
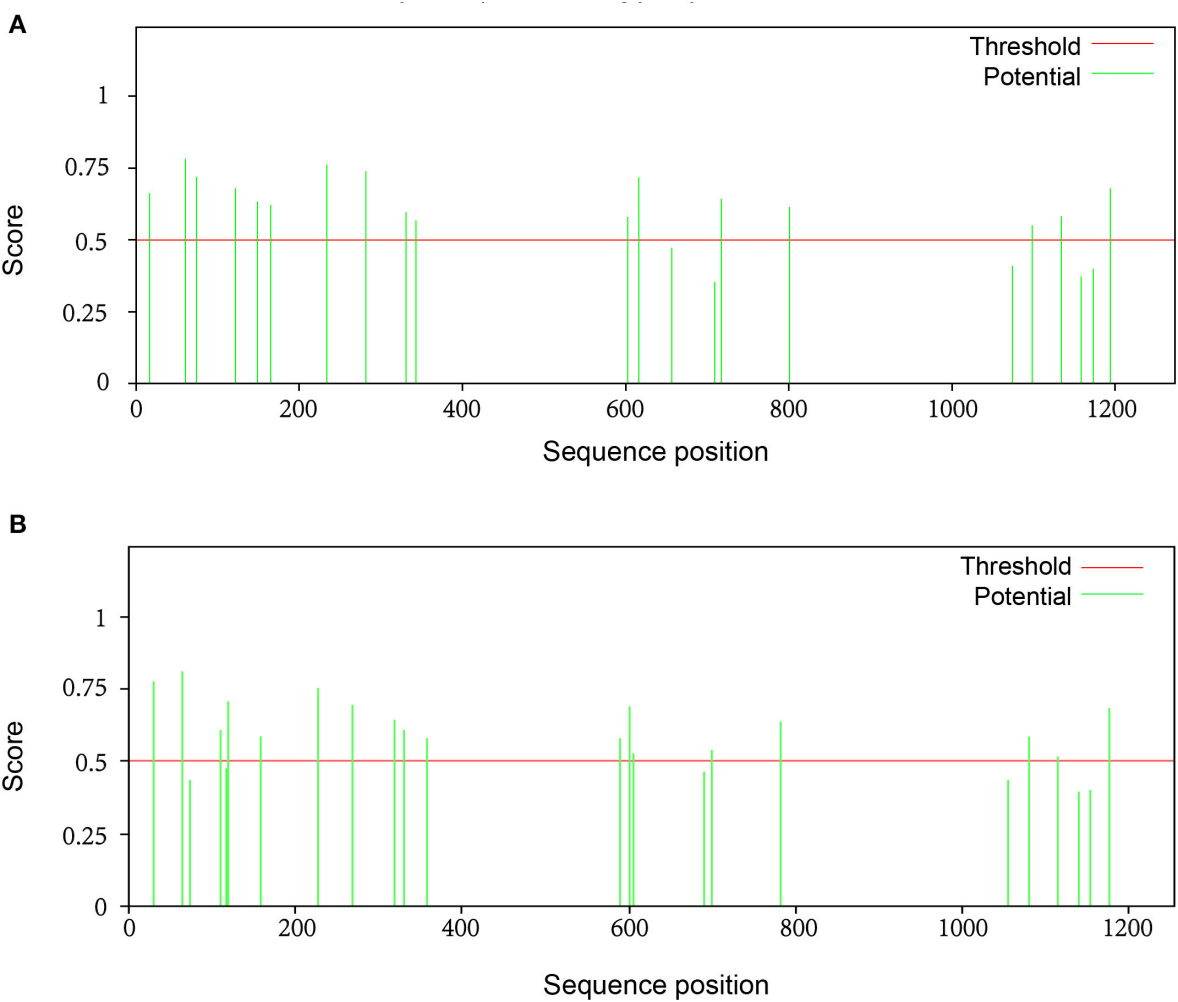
The potential N-glycosylation sites on the spike protein of SARS-CoV-2 and SARS-CoV. To identify significant differences in glycosylation patterns between SARS-CoV-2 and SARS-CoV, the potential N-glycosylation sites (green line) were predicted using the relevant web server: http://www.cbs.dtu.dk/services/NetNGlyc. There were **(A)** 22 potential N-linked glycosylation sites and **(B)** 24 potential N-linked glycosylation sites on S protein of SARS-CoV-2 and SARS-CoV, respectively. The default threshold was set at 0.5 (red line).

A total of 22 and 24 potential N-glycosylation sites were found on the S protein of SARS-CoV-2 and SARS-CoV, respectively, as predicted by the NetNGlyc web server. A total of 9 sites on SARS-CoV-2 S protein and 10 sites on SARS-CoV S protein sites were identified as N-glycosylation sites (crossing the default threshold of 0.5). The threshold and glycosylation potential of the two viruses are shown in Figure 6.

N-linked glycosylation sites on SARS-CoV-2 S protein were one less than SARS-CoV, including N61, N74, N234, N282, N616, N709, N717, N1158, and N1194 (Table 4). These results are consistent with those of other studies, even though not as many glycosylation sites were reported (18). The glycosylation sites of the two viruses were different, with no regular pattern, although the number was almost the same. Despite these gaps, the most significant difference observed was in sites N269 and N318 in the RBD region of SARS-CoV. We observed only two O-glycosylation sites on SARS-CoV S protein, with one in the RBD region, whereas three sites (S673, T678, and S686) were O-glycosylated on SARS-CoV-2 S protein (Table 4). Interestingly, these three sites flanked a polybasic cleavage site (RRAR) at the junction of S1 and S2 adjacent to the RBD.

#### Changes During the Transmission Course and Different Regions

Surprisingly, the genomic sequences collected at each month were similar from December 2019 to April 2020, indicating that no substantial variation had occurred during that time. The sequences of SARS-CoV-2 S protein did not reveal any unexpected variations, indicating the genetic stability of S protein during transmission.

In agreement with previous results, the geographical distribution of this virus was also similar all over the world. From the beginning of the outbreak at Wuhan and in other cities in China later to East Asia and part of European cities from February to March, and North America now, no significant variation was observed in the S protein sequence.

#### Structural Modeling of Glycosylation on SARS-CoV-2 Spike Protein

**Figure 7:**
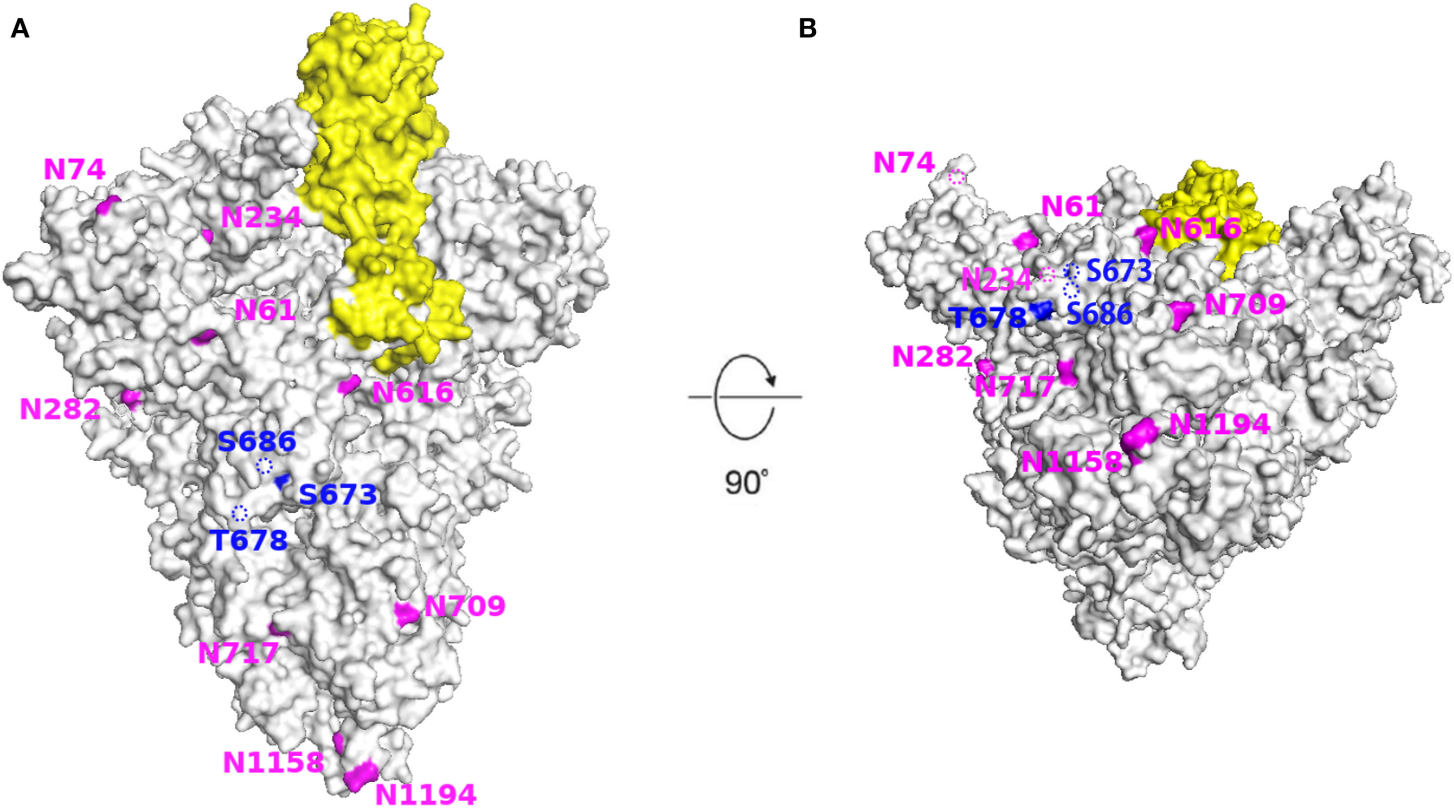
Structural modeling of SARS-CoV-2 spike protein showing the location of all glycosylation sites. The SARS-CoV-2 S protein (light gray) with receptor binding domain (yellow) and N-(amaranth) and O- (dark blue) glycosylation sites. **(A)** Side view. **(B)** Bottom view. Images generated using PyMol (version 2.3.4) based on the 3D structure (PDB ID: 6VSB). The dotted line indicates that the site is located inside the structure.

Using the cryo-EM structure of the trimeric SARS-CoV-2 S protein, we mapped the glycosylation status of the virus S protein onto the experimentally determined 3D structure (Figure 7). Our results showed that the RBD and all glycosylation sites, including N- and O-glycosylation, were clearly observed from the marked and annotated protein structure.

### Receptor Binding Domain of the SARS-CoV-2 Spike Protein

We explored the molecular structure and evolutionary pattern of the RBD, a critical element for coronavirus infection, during the COVID-19 transmission course. We observed that three amino acid substitutions within the RBD (V367F, G476S, and V483A) were identified, whose impacts in predicted cell epitope were different. Of note, G476S shortened a linear B cell epitope length and even abolished the discontinuous B cell epitope. V367F decreased the peptide binding affinity for the HLA-B35:01 allele, whereas V483A had no effect on either cell epitope. With three O-linked glycosylation sites adjacent to the domain, the evolutionary glycosylation pattern remained conserved.

## Discussion

In this study, we explored the evolutionary pattern including cell epitopes and glycosylation on the SARS-CoV-2 S protein during the global transmission course. We identified 18 S protein variations and explored their potential impacts on cell epitopes. To our knowledge, this is the first comparative study analyzing immune epitopes and glycosylation of SARS-CoV-2 variants.

According to the analysis of all sequences classified by transmission course and region, confirmed COVID-19 cases were concentrated in Asia during the early stage of epidemic, and then spread to Europe and North America. The rapid global spread of the virus provided opportunities for natural selection and to develop favorable variations facilitated transmission. Up to now, there has been limited variations on SARS-CoV-2 S protein detected, which is consistent with the results of published studies. An error-prone reverse transcription in RNA virus life circle usually drives nucleotide and amino acid substitutions in the genome, and viral evolution is also impacted by pressure from host immune response. For SARS-CoV-2, variations were also driven by this two factors, especially some amino acid substitutions had altered cell epitopes. In addition, acute transmission maybe one reason for the limited number of variations. During the pandemic, rapid transmission of SARS-CoV-2, together with lower immune response (compared with SARS-CoV) in the host may not provide enough time and selection pressure for the virus to evolve quickly.

We identified 18 relatively high frequency variants on S protein that accumulated during transmission. Among them, the D614G mutant has aroused urgent concern recently, because of its extraordinarily high frequency (16). A study suggested that the D614G mutant may have originated either in China or Europe, but spread rapidly first in Europe, and then to other parts of the world. It is now the dominant pandemic across the world (16). Another study indicated that the D614G mutant was first collected on January 28, 2020 in Germany (15). However, given the small sample size, it is hard to ascertain whether D614G is the dominant strain in these countries. Our results suggested that this variant first appeared in Zhejiang earlier (January 24, 2020) but did not become prevalent in China. Based on a recent article, the G614 variant spread faster than D614 (16). The author explained as the virus was likely to be more infectious, a hypothesis consistent with the higher infectivity observed with G614 S-pseudotyped viruses observed *in vitro*, and this variant association with higher patient Ct values, indicative of potentially higher *in vivo* viral loads. Another study reported an association between the G614 variant and higher fatality rates in a comparison of mortality rates across countries (19). Interestingly, we also found that many sequences of variants had multi-variations, of note, most of which were combined with D614G mutant. These variants, especially harboring variations would be implicated in vaccine development and antiviral therapy.

Recently, there has been published papers on the definition of B and T cell epitopes (20–22). Most defined cell epitopes on S protein were within or close to the RBD region (amino acids 319–541). Another study identified three ID sites, S370–394, S450–469, and S480–499, which were considered as T and B cell linear epitopes (23). In comparison, we identified 3 amino acid substitutions (V367F, G476S, and V483A) in the RBD region, and V483A was in one epitope. The A831V located in a defined linear B cell epitope (amino acids 818–835) was a part of the fusion peptide of S protein. Therefore, the alterations to immune response by these substitutions need further investigation. Several known coronaviruses that infect humans could trigger antibody and T cell responses in infected patients: however, antibody levels appear to wane faster than T cells. SARS-CoV-specific antibodies dropped below the detection limit within 2–3 years, whereas SARS-CoV-specific memory T cells have been detected even 11 years after SARS infection (24). An article showed that SARS-recovered patients still possess long-lasting memory T cells reactive to SARS-nucleocapsid protein 17 years. Surprisingly, they also frequently detected SARS-CoV-2 specific T cells in individuals with no history of SARS, COVID-19 or contact with SARS/COVID-19 patients (24). That indicates that T cell response may be more common and lasting than B cell response.

Many variations related to epitopes were predicted to have effects on the epitopes. Changes in the peptide length of linear B cell epitopes and discontinuous epitope conformation may affect the host immune response. The binding affinity of some epitopes switched from strong to weak binder due to specific variations (including T29I, V367F, A706V, and A831V) in context of HLA alleles. Weaker binding affinity with HLA-I usually induces lower CTL immune response in the host or even viral immune escape, and thus it is necessary to clarify whether the outcomes of patients infected with variants are different from those infected with wild types and how these variants spread during global transmission. Although a single amino acid change in an epitope may not affect recognition by antibodies, a new variant in the same epitope may emerge as the variations accumulate (25). Further experimental studies, including those on T cell and B cell, are required to determine the potential of identified epitopes to induce a positive immune response against SARS-CoV-2.

The glycosylation of viral proteins has a broad role in the field of viral physiology and pathology, including mediating protein folding and stability, and influencing viral infectivity (26, 27). The impacts of glycosylation on immune evasion have been studied for other coronaviruses (28, 29). We observed differential glycosylation in SARS-CoV-2, compared to SARS-CoV. For instance, there was no N-linked glycosylation site in the RBD region of SARS-CoV-2, whereas two sites, N269 and N318, are present in the SARS-CoV RBD region. Whether this variation is correlated with differential receptor binding affinities of the two viruses remains unclear. There were two O-glycosylation sites on the SARS-CoV S protein, one of which was in the RBD region. In contrast, there were three predicted O-glycosylation sites on the SARS-CoV-2 S protein, although none were in the RBD region. Conserved glycosylation sites during the global transmission course was another characteristic of the SARS-CoV-2 S protein. Previous researchers generated 3D structures of glycoforms of the S protein and determined the extent to which the heterogeneity of glycans impacts antigenicity (8, 30, 31). The results indicated that although glycan-heterogeneity impacted various factors (such as abundance, cell type, etc.), the efficacy of antisera does not appear to be impacted by such differences, which is consistent with our study. These results suggested that the SARS-CoV-2 S protein contains a different masking or glycan camouflage pattern compared to other coronaviruses, which may provide an evidence for the differences in host immunity.

So far, of the 3 amino acid variations identified in the RBD of SARS-CoV-2, G476S was directly in an angiotensin-converting enzyme 2 contact residue. No variation in N- or O-linked glycosylation have been observed in the RBD of SARS-CoV-2 reported, but the predicted three O-glycosylation sites (Ser673, Thr678, and Ser686) are close to each other and adjacent to the RBD. Additionally, there is a polybasic furin cleavage site (RRAR) and a leading proline inserted at the junction of S1 and S2, which allows for effective cleavage of the S protein by proteases (32). The unique structure probably impacts viral infectivity and receptor binding in the RBD. It is believed that these three predicted O-glycosylation sites can form a “mucin-like domain” to protect S protein epitopes or key residues of SARS-CoV-2 from the immune system (31, 33). Although no studies have confirmed the role of these O-linked glycosylation until now, it is likely that they make a great contribution in the immune-evasion mechanism of the virus.

Due to the various factors, such as the economic level, scientific and technological development, the number and quality of SARS-CoV-2 sequences uploaded from different countries and regions may vary widely and lack of representative. The result is probably subject to sampling biases, although it is an unavoidable objective factor. In order to mitigate this potential possibility, we downloaded all sequences (including as many countries and regions as possible) in the specified time period and excluded incomplete and low quality sequences. We analyzed all sequences available and high quality without selecting the representatives.

In conclusion, our research offers a novel perspective on the distribution characteristics of relatively high frequency amino acid variations, the impacts of T and B cell epitope variants, and the conserved evolution of glycosylation sites of SARS-CoV-2 S protein during the global transmission course. It would contribute to the evaluation of vaccine candidate immunogenicity, as well as monitoring of the potential consequences of glycosylation and cell epitope variations in the process of viral transmission.

## Data Availability Statement

Publicly available datasets were analyzed in this study. This data can be found here: GISAID database.

## Author Contributions

XZ and DY conceived of the paper. DY, WX, and MW performed the research. MW and WX designed tables and figures. WX and DY wrote the paper. XZ monitored the project. All authors contributed to the article and approved the submitted version.

## Conflict of Interest

The authors declare that the research was conducted in the absence of any commercial or financial relationships that could be construed as a potential conflict of interest.
